# Supplementary material for: Drug repurposing for aging research using model organisms
Source: Aging Cell. 2017 Jun 16;16(5):1006–15. doi: 10.1111/acel.12626 (PMC5595691; doi:10.1111/acel.12626)
Supplement: Supplementary file 7 — Data S1 Zip‐Archive of all report cards. [file ACEL-16-1006-s007.zip › RC_PY1.pdf]

# PY1

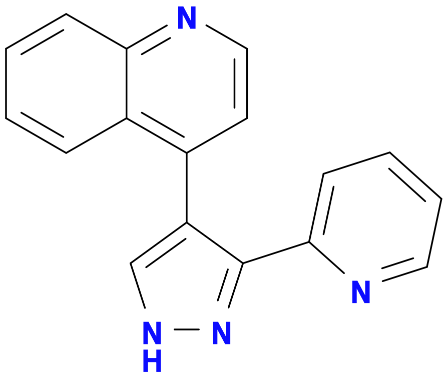

**Database identifiers**

| | |
|---|---|
| ChEMBLCompound | CHEMBL261454 |
| DrugBank | DB03921 |
| ZINC | ZINC15894680 |
| eMolecules | 36500426 |
| eMolecules | 932133 |

## Ranking

| | Rank | Score |
|---|---|---|
| Drosophila | 122/697 | 0.77 |
| C. elegans | NA | NA |

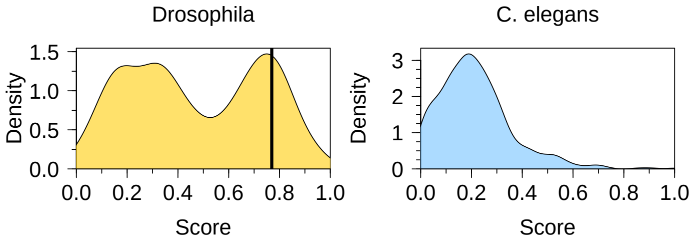


| | Ageing implication | Domain conservation | Binding site conservation | Binding affinity | Bioavailability | Lipinski | Promiscuity | Purchasability | Drug approval | **Total** |
|---|---|---|---|---|---|---|---|---|---|---|
| Drosophila | 0.792 | 0.958 | 0.962 | 0.906 | (0.9) | 0.0 | -0.0 | 0.1 | 0.075 | 0.77 |
| C. elegans | NA | NA | NA | NA | NA | NA | NA | NA | NA | NA |

## Names

- 3-(Pyridin-2-yl)-4-(4-quinonyl)-1H-pyrazole
- 4-3-(2-pyridinyl)-1H-pyrazol-4-yl-quinoline
- 4-3-(2-pyridyl)-2H-pyrazol-4-ylquinoline
- ALK5 Inhibitor I
- L6293$_{s}igmaPeakdale1_{0}$00394
- TbetaR-I Inhibitor
- TGF-beta RI Kinase Inhibitor
- Transforming Growth Factor-beta Type I Receptor Kinase Inhibitor

## Roles

ChEBI entry None has no roles

## Status

| | |
|---|---|
| Approved drug (according to ChEMBL) | No |
| Classification (according to DrugBank) | experimental |
| Number of Rule of 5 violations | 0 |
| Binding affinity to original target in log units (RF-Score prediction) | 7.27 |
| Burns *C. elegans* bioavailability prediction | 4.43 |

## Compound Target Characteristics

### TGF-beta receptor type-1

Best gene implication in ageing for this target family came from gene P80204 annotated in UniProt release 2014_02. Annotation GO 7568 (aging) was Inferred from Expression Pattern

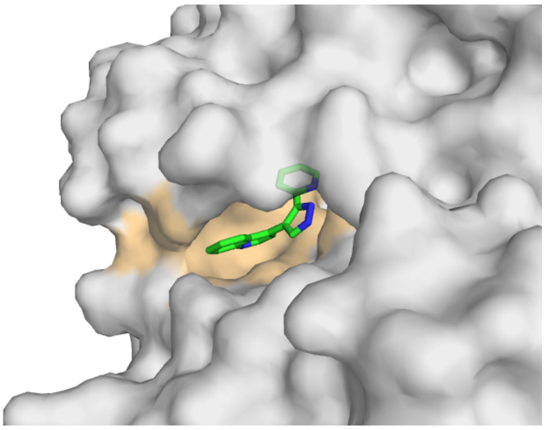

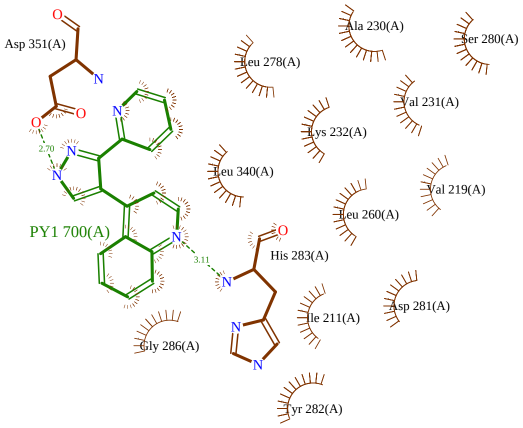


| protein | amino acids contacts (binding site) |
|---|---|
| PDB:1py5:chainA:P36897 | I V A V K L L S D Y H G L D |
| tr:F8W1R9:F8W1R9_HUMAN | I V A V K L L S D Y H G L D |
| tr:B4DY26:B4DY26_HUMAN | I V A V K L L S D Y H G L D |
| tr:Q5T7S2:Q5T7S2_HUMAN | I V A V K L L S D Y H G L D |
| sp:P36897:TGFR1_HUMAN | I V A V K L L S D Y H G L D |
| tr:Q5M9H3:Q5M9H3_RAT | I V A V K L L S D Y H G L D |
| sp:P80204:TGFR1_RAT | I V A V K L L S D Y H G L D |
| tr:Q3U485:Q3U485_MOUSE | I V A V K L L S D Y H G L D |
| tr:Q9D5H8:Q9D5H8_MOUSE | I V A V K L L S D Y H G L D |
| tr:E9Q418:E9Q418_MOUSE | I V A V K L L S D Y H G L D |
| sp:Q64729:TGFR1_MOUSE | I V A V K L L S D Y H G L D |
| tr:Q4FJL1:Q4FJL1_MOUSE | I V A V K L L S D Y H G L D |
| tr:Q7YU60:Q7YU60_DROME | I V A V K L L T D Y H G L D |
| tr:A1Z7L9:A1Z7L9_DROME | I V A V K L L T D Y H G L D |
| tr:Q23975:Q23975_DROME | I V A V K L L T D Y H G L D |
| tr:A1Z7L8:A1Z7L8_DROME | I V A V K L L T D Y H G L D |

| protein | whole protein ident | simil | domain-based ident | simil | contact-based ident | simil |
|---|---|---|---|---|---|---|
| PDB:1py5:chainA:P36897 | 1.0 | 1.0 | 1.0 | 1.0 | 1.0 | 1.0 |
| tr:F8W1R9:F8W1R9_HUMAN | 0.74 | 0.75 | 0.59 | 0.59 | 1.0 | 1.0 |
| tr:B4DY26:B4DY26_HUMAN | 0.86 | 0.86 | 1.0 | 1.0 | 1.0 | 1.0 |
| tr:Q5T7S2:Q5T7S2_HUMAN | 1.0 | 1.0 | 1.0 | 1.0 | 1.0 | 1.0 |
| sp:P36897:TGFR1_HUMAN | 1.0 | 1.0 | 1.0 | 1.0 | 1.0 | 1.0 |
| tr:Q5M9H3:Q5M9H3_RAT | 0.96 | 0.98 | 1.0 | 1.0 | 1.0 | 1.0 |
| sp:P80204:TGFR1_RAT | 0.95 | 0.97 | 1.0 | 1.0 | 1.0 | 1.0 |
| tr:Q3U485:Q3U485_MOUSE | 0.82 | 0.83 | 1.0 | 1.0 | 1.0 | 1.0 |
| tr:Q9D5H8:Q9D5H8_MOUSE | 0.77 | 0.8 | 1.0 | 1.0 | 1.0 | 1.0 |
| tr:E9Q418:E9Q418_MOUSE | 0.85 | 0.86 | 1.0 | 1.0 | 1.0 | 1.0 |
| sp:Q64729:TGFR1_MOUSE | 0.96 | 0.97 | 1.0 | 1.0 | 1.0 | 1.0 |
| tr:Q4FJL1:Q4FJL1_MOUSE | 0.96 | 0.97 | 1.0 | 1.0 | 1.0 | 1.0 |
| tr:Q7YU60:Q7YU60_DROME | 0.47 | 0.69 | 0.72 | 0.91 | 0.93 | 0.96 |
| tr:A1Z7L9:A1Z7L9_DROME | 0.46 | 0.69 | 0.72 | 0.91 | 0.93 | 0.96 |
| tr:Q23975:Q23975_DROME | 0.46 | 0.69 | 0.72 | 0.91 | 0.93 | 0.96 |
| tr:A1Z7L8:A1Z7L8_DROME | 0.45 | 0.67 | 0.72 | 0.91 | 0.93 | 0.96 |

**babo (FBgn0011300) associated phenotypes**

cell growth defective, decreased cell number, developmental rate defective, dominant, flight defective, germline clone, long lived, mitotic cell cycle defective, neuroanatomy defective, phototaxis defective, progressive, size defective, somatic clone

(Information from FlyBase)
